# Alphaherpesviruses and the Cytoskeleton in Neuronal Infections

**DOI:** 10.3390/v3070941

**Published:** 2011-06-27

**Authors:** Sofia V. Zaichick, Kevin P. Bohannon, Gregory A. Smith

**Affiliations:** Department of Microbiology-Immunology, Feinberg School of Medicine, Northwestern University, Chicago, IL 60611, USA; E-Mails: szaichik@yahoo.com (S.V.Z.); k-bohannon@northwestern.edu (K.P.B.)

**Keywords:** alphaherpesvirus, cytoskeleton, virus transport, neuroinvasion

## Abstract

Following infection of exposed peripheral tissues, neurotropic alphaherpesviruses invade nerve endings and deposit their DNA genomes into the nuclei of neurons resident in ganglia of the peripheral nervous system. The end result of these events is the establishment of a life-long latent infection. Neuroinvasion typically requires efficient viral transmission through a polarized epithelium followed by long-distance transport through the viscous axoplasm. These events are mediated by the recruitment of the cellular microtubule motor proteins to the intracellular viral particle and by alterations to the cytoskeletal architecture. The focus of this review is the interplay between neurotropic herpesviruses and the cytoskeleton.

## 1. Introduction

Herpesviruses are widespread in nature and infect a broad range of host species including vertebrates (mammals, birds, fish, reptiles) and invertebrates (mollusks, corals) [1–4]. Collectively, these viruses are categorized as the *Herpesvirales* order, with all members sharing several structural characteristics: (1) a linear double-stranded DNA genome that is typically 100 kbp or larger; (2) a T = 16 icosahedral capsid consisting of 162 capsomers (150 hexons, 11 pentons and one portal vertex); (3) an amorphous layer of tegument proteins that surrounds the capsid; (4) an envelope consisting of a lipid bilayer and membrane-bound proteins. The *Herpesvirales* are highly diverse genetically, with the most conserved protein being an ATPase that participates in packaging of viral genomes into capsids [5].

There are also similarities in key aspects of the infectious cycle, which include: (1) entry into the cell via receptor-mediated fusion, (2) delivery of the encapsidated genome to the nucleus, (3) establishment of life-long latent infection, (4) DNA replication and capsid assembly inside the nucleus, (5) exit of encapsidated genomes from the nucleus to the cytosol, (6) maturation and envelopment of viral particles in the cytoplasm, and (7) exocytosis of infectious viral particles.

The importance of interactions between viruses and the host cytoskeleton is well established in cultured systems [6–10]. However, the mechanism of certain viral-induced cytoskeletal changes and the benefit they convey to the replicating virus remain unclear. Moreover, differences between culture models of infection and how cytoskeletal interactions impact infection in animal hosts need to be addressed. This review summarizes our current knowledge of alphaherpesvirus engagement of the host cytoskeleton and discusses the implications of these processes to neuroinvasion.

### 1.1. The Alphaherpesvirinae

**Figure 1:**
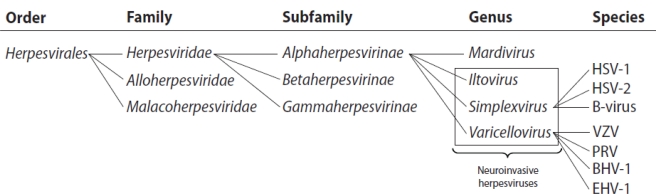
Taxonomy of neuroinvasive herpesviruses. Neuroinvasive herpesviruses include species from three genera of the *Alphaherpesvirinae* subfamily. Virus species mentioned in this review are as follows: herpes simplex virus type 1 (HSV-1) and type 2 (HSV-2); monkey B virus (B-virus); varicella-zoster virus (VZV); pseudorabies virus (PRV); bovine herpesvirus type 1 (BHV-1); equine herpesvirus type 1 (EHV-1).

Although all herpesviruses share several important biological characteristics, they vary largely in host range, tissue tropism, replication kinetics, clinical symptoms and disease severity [11,12]. There are three families within the *Herpesvirales* order of which one, the *Herpesviridae*, includes known pathogens of mammals. The *Herpesviridae* is further divided into three subfamilies named the *Alpha-*, *Beta-* and *Gammaherpesvirinae* [13–15]. The neuroinvasive herpesviruses, which are the subject of this review, are members of three of the four genera of *Alphaherpesvirinae*, two of which include mammalian and marsupial pathogens: the *Simplexviruses* and the *Varicelloviruses* (Figure 1) [13]. The term “neuroinvasion” can have differing applications: in this review we apply the term to refer to invasion of neurons that were not directly exposed to inoculation (*i.e.*, spread from non-neuronal tissue to neurons), however, the term may also specifically refer to invasion of neurons resident in the central nervous system (*i.e.*, encephalitic infections).

A common characteristic of the *Alphaherpesvirinae* is a rapid reproductive cycle, followed by destruction of the host cell in a wide variety of susceptible tissues [16]. Although infections are often not associated with evident symptoms, severe disease (for example encephalitis and meningitis) can arise from the active replication of these viruses coupled with their propensity to spread within neural circuits. This is in contrast to the lymphotropic *Gammaherpesvirinae* which, in general, produce severe disease (for example lymphomas and sarcomas) as a consequence of latency [17]. The prototype member of the *Simplexvirus* genus is *Human herpesvirus 1* (herpes simplex type 1; HSV-1). Other human pathogens in this family are *Human herpesvirus 2* (herpes simplex type 2; HSV-2) and *Cercopithecine herpesvirus 1* (B-virus). Although the latter virus is not endemic in humans, zoonotic spread can result in lethal infections [18]. The prototype member of the *Varicellovirus* genus is *Human herpes virus 3* (varicella-zoster virus; VZV), which is the causative agent of chickenpox and shingles. This genus also encompasses a number of veterinary pathogens, including *Suid herpesvirus 1* (pseudorabies virus; PRV), which is commonly used to model neuroinvasive infections both *in vitro* and *in vivo*. The remaining two genera, *Mardiviruses* and *Iltoviruses*, comprise viruses of avian tropism that are classified as *Alphaherpesvirinae* primarily based on genetic conservation [13]. Like viruses belonging to the *Gammaherpesvirinae* subfamily, the *Mardiviruses* establish latency in T lymphocytes and are noted for their oncogenicity [19]. Although *Mardiviruses* have not been detected in neurons *in vivo*, they infect explanted non-myelinated neurons and surrounding Schwann cells. However, demyelination of peripheral axons is more likely caused by virus-induced T lymphocyte infiltration and not direct neuronal infection [20]. The *Iltoviruses* cause contagious respiratory disease. Chickens infected with the prototype of this genera, *Gallid herpesvirus 1* (infectious laryngotracheitis virus; ILTV), test positive by PCR for infection of the trigeminal ganglia. The virus is therefore considered neuroinvasive, although confirmation of infection of neurons within the ganglia is not available [21].

### 1.2. Neuroinvasive Herpesvirus Disease

HSV-1 and HSV-2 symptoms are usually restricted to epidermal lesions around the mouth (cold sores; herpes labialis) and genitals, respectively. Less frequently, HSV-1 infects the eye and produces keratitis, which is the leading cause of infectious blindness in the developed world [22]. Each of these forms of disease can reoccur due to the latent infection that is established in neurons of the peripheral ganglia that innervates the respective tissues (typically the trigeminal ganglia for HSV-1 and the lumbosacral dorsal root ganglia for HSV-2). Autonomic ganglia also may become infected [23,24]. Rarer but more severe, HSV-1 transmission from the peripheral nervous system to the brain can produce life-threatening encephalitis. Herpes encephalitis is generally sporadic and infrequent, although there is evidence for genetic predisposition to this severe form of disease [25]. Herpes encephalitis is most frequent in neonates infected during birth and these infections can also produce multi-organ disseminated disease, both of which are associated with high mortality and morbidity [26]. Adults with compromised immune systems (including patients with AIDS, cancer, and transplant patients) can suffer disseminated infections that become resistant to acyclovir [27,28].

Unlike the *Simplexviruses*, primary VZV infection (chicken pox; varicella) initially disseminates by cell-associated viremia following replication in lymph nodes. Subsequently, the virus expands after additional replication in the liver and spleen. Thus, VZV has broad tissue tropism that includes T-cells and monocytes. The infection subsequently transmits to the skin where virus-driven cell-cell fusion induces polykaryocyte formation and characteristic pox lesions [29]. At this stage of the infection, virions in the skin invade the sensory nervous system in a manner similar to *Simplexviruses*, and establish latency in neurons of the dorsal root ganglia. Reactivation of VZV is usually infrequent and is often in a single dorsal root ganglion of an older individual, possibly due to waning immunity to the virus. Each dorsal root ganglion innervates a specific region of the skin (a dermatome), which defines the presentation area and size of the resulting painful skin lesions (shingles; zoster) [30]. The symptoms of pain can persist long after the rash has receded in a manifestation termed postherpetic neuralgia. Like the *Simplexviruses*, neuroinvasion by VZV is usually confined to the PNS; spread to the CNS is rare but severe. VZV infection can also cause life-threatening diseases, including encephalitis and pneumonia, in individuals with compromised immunity [31,32].

A wide assortment of neuroinvasive herpesviruses infects domestic and wild animals. Common presentations include skin lesions, respiratory disease, keratitis, abortion, pneumonia, and encephalitis [33–35]. Some of these viruses have a propensity to jump to secondary hosts, which typically result in lethal, dead-end infections. Unlike infections of natural hosts, in which neuroinvasion is typically restricted to the peripheral nervous system, dead-end host infections spread to the brain by default. Perhaps the most striking example of this is PRV, which normally resides in pig populations (the natural host) but transmits to mammals ranging from rodents to large carnivores [36]. Similarly, B-virus transmission to humans is fatal if left untreated [37]; and conversely, marmosets are susceptible to lethal HSV-1 infections [38]. Remarkably, CNS invasion occurs by trans-synaptic transmission along neuronal circuits: these viruses are highly proficient at spreading between neurons across synaptic connections even though this attribute provides no clear selective advantage to these viruses [39,40].

## 2. Alphaherpesviruses and the Host Cell Cytoskeleton: Interactions and Virus-Induced Changes

Alphaherpesviruses, like all viruses, are obligate intracellular parasites that depend on many aspects of the cell to propagate and ultimately maintain their presence in host populations. Gaining access to the resources of the cell requires specialized adaptation. Here we discuss a selection of tactics used by neuroinvasive alphaherpesviruses to subvert innate cellular transport mechanisms and effect viral replication and spread within cells and tissues.

### 2.1. Cytoskeleton Organization of Polarized Epithelium and Sensory Neurons

Neuroinvasive alphaherpesviruses initially replicate in epithelia and subsequently establish latency in the peripheral nervous system, predominantly in pseudounipolar neurons resident in sensory ganglia. A morphological and functional hallmark of both epithelial cells and neurons is their polarity, and similarities between the establishment and function of the polarity in these two cell types have been noted. For example, proteins that sort to the apical membrane of epithelial cells often sort specifically to axons when expressed in neurons [41,42], and epithelial adherence junctions and neuronal puncta adherentia junctions are likely related structures [43–45]. However, for the most part these cells are unique, with differences in the organization and maintenance of the cytoskeletal network defining the structural and functional polarity of each cell type. As will be discussed, differences in cellular architectures present an obstacle for herpesviruses: polar cells require polar modes of viral entry and egress that must be adjusted between epithelial and neuronal cells. The herpesvirus infectious cycle is achieved by recognizing and adjusting to different intracellular environments. Regulation of the resulting life-long infection in stages of productive and latent infection adds further complexity to the infectious cycle to restrict pathogenesis and maximize dissemination in host populations. This is an impressive feat that helps explain why the majority of the world’s population is infected by these viruses, with one critical element of this success arising from the interactions between these neuroinvasive viruses and the cellular cytoskeleton.

The cytoskeleton is a dynamic assembly of structures that facilitates changes in cell shape, cell locomotion, chromosomal segregation, cell division, and intracellular trafficking of molecular complexes and organelles [46,47]. The cytoskeleton of mammalian cells consists of three main components: actin, tubulin and intermediate filaments. Each of these exists in a dynamic equilibrium between monomeric and filamentous forms. Although detailed descriptions of the biology of each of these cytoskeletal elements are widely available, we will next present brief overviews of the F-actin and microtubule cytoskeletal components found in epithelial and neuronal cells with an emphasis of those features most relevant to the subsequent discussion of neuroinvasive herpesvirus infection.

#### 2.1.1. Actin

The actin monomer is an ATPase, with ATP hydrolysis driving the dynamic balance between monomeric G-actin (globular) and filamentous F-actin in the cell [48]. Actin filaments have a 7–8 nm diameter and are intrinsically polar, with the barbed/plus ends often associated with the plasma membrane and exhibiting high turnover [49]. In non-muscle cells, actin filaments build a densely branched mesh underneath the plasma membrane and provide the driving force for membrane ruffling, lamellipodia and filopodia outgrowth, and macropinocytosis. The highly dynamic nature of actin filaments allows for rapid responses to extra- or intra-cellular cues [50]. In non-musculature cells, actin filaments build a dense mesh underneath the plasma membrane. Regulation of these dynamics is achieved by an extensive array of actin-associated proteins including small GTPases [51–53], and by posttranslational modification of different actin isoforms including direct phosphorylation by PAK1 (p21 protein (Cdc42/Rab)-activated kinases 1) [54,55].

The polarized structure of epithelia is dependent upon actin. The apical section of an epithelial cell contains three zones of actin filaments that include: filament bundles that support microvilli, filaments connected to tight junctions, and a perijunctional belt of actin filaments that directly binds catenin/cadherin and afadin/nectin adhesion complexes [56,57]. This specific organization of actin is essential for establishment and maintenance of apical-basal polarity and maintenance of cell-cell adhesion.

In neurons, polarity is established by actin dynamics that drive the outgrowth of axons within a lamellipodial structure called the growth cone [58]. Upon maturation, the axon growth cone is replaced by a specialized axon ending, which may consist of a presynaptic terminal that communicates with a muscle cell or another neuron or a sensory nerve ending such as those in the skin and mucosa. Maintenance of neuronal polarity is primarily the purview of microtubules and intermediate filaments; however, actin continues to contribute to polarity by providing a selective barrier function within the axon hillock. The hillock forms the soma-axon junction and keeps the Golgi apparatus and lysosomes inside the cell body while allowing for the passage of mitochondria and pre-synaptic vesicles into the axon [59]. Actin also plays major roles in the morphogenesis of synapses, including presynaptic terminals, post synaptic dendritic spines, and puncta adherentia junctions (PAJ) that strengthen synaptic connections through interactions between presynaptic nectin-1 and postsynaptic nectin-3 [51]. Synaptic vesicle endocytosis and exocytosis at presynaptic terminals is also modulated by actin dynamics [60].

#### 2.1.2. Tubulin and Microtubules

Tubulin dimers assemble into 25 nm tubular structures called microtubules that provide a network for long-distance intracellular transport [61]. Similar to actin filaments, microtubules are polar structures with a dynamic plus end and a more stable minus end. Regulation of microtubule dynamics is modulated by microtubule-associated proteins and small GTPases [61]. Moreover, tubulin is posttranslationally modified in a number of ways, including by phosphorylation, which affects the stability of microtubule and the repertoire of interactions with microtubule-associated proteins [61]. Microtubule organization is typically illustrated as a radiating array of filaments that emanates from a single perinuclear centrosome that serves as the microtubule organizing center (MTOC) with dynamic plus ends projecting to the plasma membrane and minus ends anchored to gamma tubulin within the centrosome. However, this topology does not accurately document the microtubule arrays found in polarized cells. In many cell types microtubules are released from the MTOC and increasing evidence suggests that gamma-tubulin-dependent microtubule nucleation does not have to be connected with a conventional MTOC [62,63].

**Figure 2:**
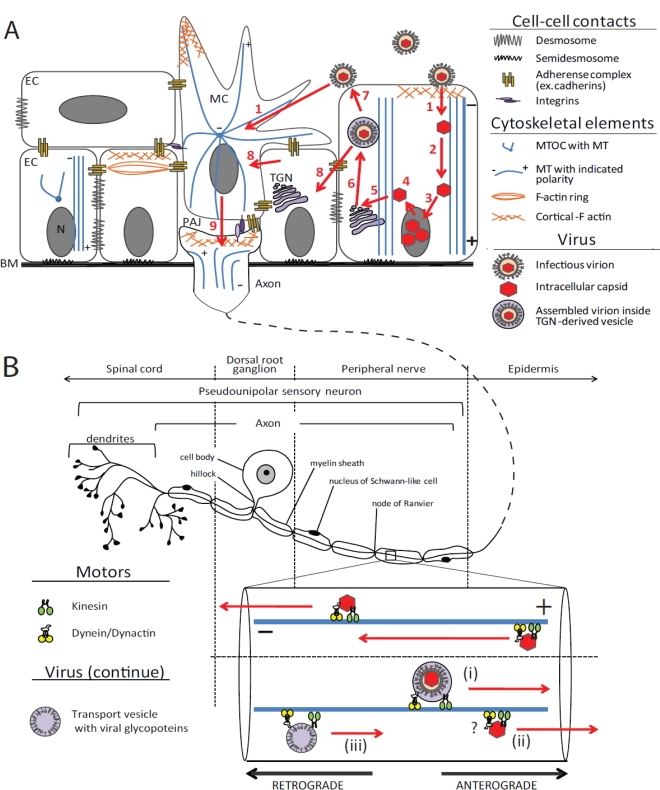
Alphaherpesvirus intracellular transport, dissemination, and neuroinvasion. (**A**) Alphaherpesviruses replicate in epithelia, which have complex multilayer architectures. The epidermis has multiple cell types, including polarized cells (epithelial cells; EC) adjacent to the basement membrane (BM) which have microtubule bundles with minus ends oriented toward the apical surface. On top of the polarized layer may be multiple layers of epithelial cells. Merkel cells (MC) form punctae adherentia junctions (PAJ) with nerve fibers of sensory neurons and have a classic microtubule aster emanating from the microtubule organizing center in the perinuclear region. Viruses enter cells by receptor-mediated fusion (1), followed by microtubule-based transport of the capsid and a small number of capsid-associated tegument proteins (2). The nucleus is the final destination of the virus (3). Newly assembled viral particles exit the nucleus and the resulting naked capsids (4) are delivered to the TGN for final assembly (5). Inside the TGN-derived vesicles, assembled virions are transported to the plasma membrane (6) where they exocytose (7). Virions can spread to the neighboring cells via cell-cell contact sites (8). Neuroinvasion may occur across the PAJ (9), following basal release of virions from EC in proximity to the nerve terminal, or by infection of exposed nervous endings. (**B**) Sensory neurons are a primary target of alphaherpesvirus virions. A part of the axon is magnified to show the architecture of the transporting virus particles, which depends on the directionality of transport. Retrograde-transporting particles consist of the capsid and associated tegument proteins. Viral particles arising from reactivated infection primarily transport as fully-assembled virions inside vesicles (i); however virus components could be transported independently of each other (ii, iii; denoted with a question mark—see text). Viral particles at both stages of infection associate with kinesin and dynein motors.

In polarized epithelia, microtubules are organized in a non-centrosomal array aligned along the apical-basal polarity cell axis with minus ends apical and plus ends basal (Figure 2A) [64]. Anchoring of microtubule minus ends can occur at the most apical part of the cadherin-based adherens junction, called the zonula adherens [65]. Additionally, there is a meshwork of microtubules beneath the apical membrane and longitudinally arranged microtubule bundles around the nucleus at the lateral membrane (with plus-ends facing the basal surface of the cell) [66]. This unique microtubule organization is established as epithelial cells polarize and provides for transcytosis and trans-epithelial intracellular traffic flow.

The axons of neurons contain tracts of parallel microtubules with plus ends facing the distal axon and minus ends oriented toward the soma [67]. In a pseudounipolar sensory neuron, the axon bifurcates near the soma, with the peripheral branch extending to the epidermis and the central branch to the spinal cord. Although the peripheral branch of the axon functions as a dendrite from an electrophysiological perspective (information flows toward the soma), it is myelinated and has a microtubule architecture that is axonal (plus ends are uniformly oriented toward the axonal terminus) (Figure 2A) [68]. Axonal microtubules are stabilized by a number of neuron-specific microtubule-associated proteins including tau-like proteins and STOP proteins (stable tubulin-only polypeptide) [67].

#### 2.1.3. Molecular Motors

Facilitated intracellular transport is mediated along F-actin and microtubules by large molecular motor complexes that move cargoes by hydrolyzing ATP. The myosin family consists of actin-based motors that participate in muscle contraction and actin-based transport (processive myosins) [69]. There are several classes of myosins: myosin I isoforms are membrane-associated and affect membrane dynamics, cytoskeletal structure, mechanical signal transduction and endosome processing; myosin II is the major contractile motor in muscles but also contributes to the dynamics of cortical actin and actin belts that connect adhering junctions [70]. Two major myosin II non-muscle isoforms play different roles: myosin-IIB participates in tension maintenance (actomyosin-generated force combined with linkage to the cell surface receptors that is responsible for adhesion [71]) [72,73], whereas myosin-IIA isoform is more suited for F-actin sliding (anti-parallel sliding of actin filaments) due to its low affinity binding to actin [74]. Myosin IIA is proposed to cross-bind actin fibers with microtubules and restrain cell migration [75]. The absence of myosin IIA results in stabilization of microtubules, suggesting a role for myosin IIA in regulating a balance between the actomyosin and microtubule systems [75]. The last two major classes of myosins are myosin V and myosin VI, both noted for their processivity and contribution to vesicular transport [70].

The kinesin superfamily (KIF) of molecular motors consists of over 250 kinesin-like proteins. Kinesins have a common fold and ancestry with myosins and G proteins [76]. The majority of kinesins work as a hetero-complex with non-motor proteins called kinesin light chains (KLCs). The kinesin superfamily is currently divided into 15 groups [77,78]. Kinesin-1 (conventional kinesin group), -2, -3, -4, -12, and -14 families consist of processive cytoplasmic kinesins that participate in vesicle and organelle transport. Kinesins from other families are required for spindle formation/maintenance, chromosomal segregation, kinetochore capture and microtubule dynamics. The kinesin-1 group contains KIF5A, KIF5B and KIF5C isoforms, where KIF5A and KIF5C are neuron-specific and KIF5B is ubiquitously expressed [79,80]. Transport in neurons requires a differentiation between axonal and dendritic transport as well as vesicle sorting, and several kinesins contribute to maintaining neuron polarity and function. KIF1A and KIF1Bβ, kinesin-3 family members, are axon anterograde transporters of Rab3-containing presynaptic vesicles [81,82]. Neuronal kinesin-14 motors (KIFC2 and KIFC3) are unusual minus-end directed C-type kinesins that are proposed to play a role in retrograde transport in dendrites and axons [83–85]. KIF17 (a kinesin-2 family member) is a dendrite-specific anterograde motor and is implicated in transporting *N*-methyl-d-aspartate receptor NR2B subunit-containing vesicles associated with glutamatergic neurotransmission [86]. Another interesting neuron-specific kinesin is KIF3C, also a kinesin-2 family member, binds and transports Fragile X mental retardation 1 protein (FMRP), an RNA-binding protein, to local sites of protein synthesis in axons and dendrites [87]. KIF3C binds KIF3A, another kinesin-2 family member, and forms a heterogenic, neuron-specific, vesicle-associated motor protein complex [88]. Further diversity of the kinesin families is provided by numerous types and isoforms of associated KLC. For example, KIF5B can associate with various isoforms of KLC1 or KLC2 that arise from alternative splicing [89].

Unlike kinesins, most mammals have only 3–5 genes encoding the predominant minus-end directed microtubule motor: Dynein. While the diversity of plus-end microtubule trafficking is conveyed by the large superfamily of kinesin motors, minus-end trafficking is coordinated by interactions of different regulatory proteins with the dynein motor. Heterogeneity in the dynein complex dictates motor stability, processivity, and cargo selectivity [90,91]. The dynein motor complex is the largest and most complex of the three classes of cytoskeletal motors: its structure consists of two heavy chains, three intermediate chains and four light chains. Heavy chains have a motor domain unlike those of kinesins and myosins, having homology with the AAA superfamily of mechanoenzymes [92]. Additionally, dynein typically functions in conjunction with the dynactin protein complex, which consists of at least ten additional proteins including: actin related protein 1 (Arp1), p150 (glued) and p50 (dynamitin) [93].

Each cytoskeletal motor has a specific directionality. Myosins I and V move towards the barbed/plus end of the actin filaments, while myosin VI moves in the opposite direction [94,95]. Most processive cytoplasmic kinesins move toward the plus end of the microtubule with the exception of members of the kinesin-14 group. The majority of minus-end directed motion along microtubules is achieved by the dynein/dynactin complex [96]. Thus, all traffic in non-polar mesenchymal cells from the plasma membrane inward toward the nucleus is dynein-dependent, whereas kinesins transport cargoes toward the plasma membrane. Because polarized epithelial cells have microtubules spanning the cell with minus ends apical and plus ends basal, all transport toward the basolateral membrane is kinesin-based and transport in the apical direction is achieved via dynein [97,98]. In general, cargo transport switches from microtubules to F-actin near the cell periphery where myosins I, V and VI take over [99].

Interestingly, kinesin and dynein can work independently [100], cooperatively [100–102], or competitively [103]. In addition, kinesin-1, dynein and myosin can form a heterocomplex that moves on both F-actin and microtubules [104]. The dynactin complex plays a key role in bi-directional movement by promoting both dynein [105–107] and kinesin transport [107,108]. Moreover, p150^glued^ as well as another known dynein-binding protein (BICD2) can crosslink dynein and kinesin motor protein complexes and thus regulate bidirectional transport of vesicles and positioning of the nucleus [109,110]. In some circumstances, kinesin and dynein can interact with each other directly [111].

Cytoskeletal motor proteins are regulated by phosphorylation and by phosphorylation of associated proteins [112,113], and, in the case of myosins, are also regulated by calcium flux [98,114]. Myosins are also known to be regulated via ubiquitination of motors themselves [115], or myosin chaperone proteins [116]. Tubulin and actin are also substrates for ubiquitination [54,117]. Additionally, motor protein regulation occurs at the level of cargo binding [112]. Within this complexity of transport regulation, it is important to note that microtubule- and actin-dependent transport are often coupled and coordinated [112,118]. While the big picture of cytoskeletal-based transport is far from complete, in general, the coordination of trafficking events and targeting of cargo delivery is modulated by coupling specific motors and cargo, adjusting motor activity, and transporting along cytoskeletal tracks that are themselves dynamic.

### 2.2. Neuroinvasive Herpesviruses and the Cytoskeleton

Herpesviruses interact with, and sometimes rearrange, the cellular cytoskeleton. These interactions occur throughout the infectious cycle: From extracellular viral particles attached to the surface of cells, viral structural components deposited into the cytosol upon initial entry, and *de novo* expressed viral proteins in the cell. Therefore, herpesvirus envelope proteins, tegument proteins, capsid proteins and non-structural proteins each have opportunities to enhance infection by subverting cytoskeletal function.

#### 2.2.1. Virus Entry: Initiation of Cytoskeleton Rearrangements

Neuroinvasive herpesviruses initially attach to cells by binding heparin sulfate (HS) on heparin sulfate proteoglycans (HSPGs). For HSV-1, binding to HS can be mediated by gC and, to a lesser extent, by gB [119–121]. Virion binding to cell surface HS is thought to promote subsequent interactions with herpesvirus entry receptors by retaining virions at the plasma membrane. However, this conceptually passive virion retention mechanism also triggers a burst of signals into the virion and cell. HSV-1 virions undergo a structural deformation upon binding to cells that includes release of a tegument protein, pUL16, from the capsid. The effect this change has on subsequent viral entry is unclear; however, gC binding to HS is sufficient for its induction [122]. Meanwhile, HSPGs bound by gC, as shown for PRV, relocalize within the plasma membrane and become aligned with the underlying actin cytoskeleton [123]. In this regard, the virus appears to have co-opted a normal cellular function of HSPGs, which serve as a low-affinity receptors for many growth factors, align to the actin cytoskeleton and become insoluble to Triton X-100 upon growth factor binding [124]. This transmembrane association with the actin cytoskeleton is thought to effectively deliver the bound growth factor, or herpes virion as the case maybe, to its high-affinity receptor.

Viral attachment to the cell surface can occur along finger-like protrusions referred to as filopodia. Attachment of HSV-1 to filopodia via gB-HS interaction connects virions to the retrograde actin flow, bringing them to the filopodia base at the cellular membrane [125]. This process, referred to as “viral surfing” was originally documented for retroviruses, but is increasingly recognized as a common viral tactic to increase the efficiency of entry for both enveloped and non-enveloped viruses [126]. In cells plated at low density in culture filopodia increase the cell surface area, thereby increasing the number of virions that enter a cell independently of the multiplicity of infection (MOI). Formation of filopodia depends on activation of Cdc42, a Rho GTPase [127]. For HSV-1, binding of cell surface nectin by gD can deliver this signal, while inhibition of phosphoinositide 3 kinase (PI3K) antagonizes the gD signal and filopodia formation [128–130]. Therefore, viruses do not only surf but also induce the filopodia upon which surfing occurs. Inhibition of PI3K does not interfere with HSV-1 attachment or entry but does prevent incoming capsids from arriving at the nucleus [131]. Together, these findings suggest that HSV-1 surfing may direct incoming virions to productive sites of viral entry, consistent with a suggestion that surfing helps to avoid the delivery of capsids into dense actin filament regions of the cortex that may confine the capsid and hinder subsequent intracellular transport [126]. Although HSV-1 delivered to cells at exceedingly high MOI can induce filopodia formation [125], the initiation of this process may be more relevant in the context of cell-cell transmission of infection. Herpes glycoproteins are expressed on the surface of infected cells, making them accessible to HS on neighboring cells [132]. The triggering of Cdc42 in a target cell would likely establish a dynamic cell-cell junction that efficiently recruits emerging virions to the uninfected cell, functionally related to the virological synapse induced in T cells by HIV-1 [133] and notably by HSV-1 [134]. Perhaps related to the induction of filopodia, binding of gD to sensory neurons elicits a Cdc42 signal that results in the formation of axon varicosities, which are sites of herpesvirus entry and exit in neurons [135]. A related finding is seen in CNS neurons, which respond to gD binding by increasing synapse number on axons [45]. A general theme is therefore emerging that herpesvirus glycoproteins not only mediate virion entry, but also remodel both neurons and non-neuronal cells by triggering actin dynamics through Cdc42 to make the cells more permissive to infection.

Herpesvirus surfing and internal virion rearrangements represent exciting new areas of research, yet these events are dispensable for infection; pUL16, gC, and the HS-binding activity of gB are all non-essential for HSV-1 propagation in culture [136–141]. Furthermore, the role of the actin cytoskeleton in herpesvirus entry is variable. HSV-1 entry into HEp-2 cells and PRV entry into PK15 cells are sensitive to actin depolymerization with cytochalasin D [123,142], while cytochalasin D treatment of Vero cells does not impact HSV-1 or PRV entry [123,143]. Nevertheless, HS interactions are essential both for efficient attachment and entry [138]. These facts likely speak more to the limitations of studying viral infections in transformed tissue culture cell lines than they do about the relevance of the processes themselves.

In neurons, HSV-1 enters by envelope fusion with the plasma membrane, typically at axon terminals [144–146]. The capsid must therefore navigate to a microtubule to engage in retrograde transport. Whether this is a passive or active process is not known. In some cell types, HSV-1 fusion occurs after endocytosis [144,147,148]. Engagement of virion gD by cellular nectin or HVEM is generally a prerequisite for fusion, but gD cannot be the only trigger for fusion as this protein is absent from VZV and dispensable for PRV cell-cell transmission [149–152]. While far less is known regarding alternative entry receptors, their potential roles in cytoskeletal interactions makes them worthy of mention here. HSV-1 gB binds paired immunoglobulin-like type 2 receptor alpha (PILRα) [153,154], and non-muscle myosin IIA heavy chain IIA (NMHC-IIA) [155]. PILRα is one of two paired antagonistic receptors (PILRα is inhibitory and PILRβ is stimulatory) mainly expressed on myeloid cells such as monocytes, macrophages, and dendritic cells, and plays an important role in regulation of immune cells [156,157]. The role of PILRα in HSV infection *in vivo* is not known, however, it is possible that PILRα, along with HMEV and nectin-1, plays a role in HSV-2 entry into retinal pigment epithelial [158]. Contractile myosin IIA, a major participant in control of cell adhesion, migration, and tissue architecture, is an unusual receptor for HSV entry, primarily because of its cytosolic localization. However, NMHC-IIA was redistributed and accumulated at the plasma membrane 2–15 min after HSV-1 infection. Moreover, NMHC-IIA was reported to become cell-surface exposed, allowing for its interaction with HSV gB to promote virus-cell fusion. Knockdown of NMHC-IIA or blocking NMHC-IIA with antibody inhibited HSV infection [155]. Furthermore, NMHC-IIA subcellular redistribution depends on phosphorylation by Ca^2+^-calmodulin-dependent myosin light chain kinase and specific inhibitor of this kinase (ML-7) or chelation of Ca^2+^ decreased Vero cell susceptibility to HSV-1 [155]. It is noteworthy that virus surfing is dependent on myosin II, and inhibition of myosin II with blebbistatin significantly reduces viral infection [126,159].

VZV gB, gH/gL and HSV-1 gB were recently shown to interact with myelin-associated glycoprotein (MAG) [160]. MAG is a component of myelin in the CNS and PNS and promotes neurite outgrowth during embryonic development. MAG can form a complex with β1-integrin and promote axonal growth by modulating cytoskeletal dynamics, cell adhesion, and migration [161].

Following entry, Cdc42 signaling appears to provide a second enhancement of viral infectivity. Phosphorylation of Cdc42 leads to activation of the related GTPase, RhoA, and activation of this temporal Cdc42/RhoA cascade is observed during HSV-1 infection [127]. RhoA can effect changes in the actin cytoskeleton through Rho-associated coiled-coil containing protein kinase 1 (ROCK1), which in turn signals to focal adhesion kinase (FAK) [162,163]. Both ROCK1 and FAK are implicated in HSV-1 and EHV-1 infectivity downstream of entry [164,165]. These observations are consistent with the finding noted earlier that PI3K inhibition also blocks infection at a post-entry step [131]. For EHV-1, this may result in productive caveolin-dependent endocytosis and subsequent intracellular transport that is dependent on depolymerization of F-actin and intact microtubules [166,167]. Activation of Cdc42 leads to stabilization of the microtubules [168] that, by providing a route for capsid transport to the nucleus, may provide one mechanism for enhanced permissivity of herpesvirus infection following entry.

#### 2.2.2. Transport of Viral Particles in the Cytoplasm

Upon entry, membrane fusion deposits the capsid in the cytosol and leaves glycoproteins and the envelope behind as a contiguous part of the cellular membrane [144–146,169–174]. Tegument proteins have a mixed fate during this disassembly step. Tegument proteins pUL36 (VP1/2), pUL37 and pUS3, remain capsid bound and are constituents of the capsid-transport complex that ultimately docks at nuclear pores, while other tegument proteins disassociate from the capsid [175–178]. Long distance transport of capsids (*i.e.*, from the site of entry to the nucleus) occurs along microtubules (Figure 2A and 2B) [143,179]. In most cells minus-end directed transport along microtubules moves capsids toward the nucleus, and is termed retrograde. Conversely, anterograde transport is directed towards microtubule plus ends and moves capsids toward the plasma membrane. In neurons, retrograde transport is responsible for moving capsids from axon terminals to the neuronal cell body, while reactivated herpesvirus infection undergo anterograde transport from the cell body to axon terminals. A notable exception to this rule is observed in polarized epithelia, which have longitudinal microtubules extending lengthwise within the cell and oriented with minus-ends apical and plus-ends basal (Figure 2A) [66,180–182]. Both apical and basolateral entry of HSV-1 into polarized epithelial cells (MDCK) was reduced when cells were treated with nocodazole (a microtubule-depolymerizing drug), implicating microtubules in capsid transport from both directions [183]. Transport of alphaherpesviruses along microtubules is dependent upon cellular microtubule motor complexes, which are too large to be encoded by a herpesvirus, to achieve these transport events. Although selective depolymerization of microtubules with nocodazole demonstrates that the microtubule cytoskeleton is not essential for herpesvirus infection in transformed cell lines [143,183], passive diffusion is not sufficient to deliver herpesvirus capsids from an axon terminals to neuronal cell bodies [68,179]. Therefore, microtubule-based axon transport is a critical factor in alphaherpesvirus neuroinvasion, the establishment of latency, and reactivated infection.

##### 2.2.2.1. Retrograde Transport: Ingress

The first obstacle that viral particles encounter on the way to the nucleus is a thick layer of cortical actin. As discussed, interactions of virus with the cell surface receptors are expected to initiate Cdc42-dependent rearrangement of the actin cytoskeleton. It is not known, however, if rearrangement of actin is required for early stages on alphaherpesvirus infection *in vivo*. In Vero cells and cultured sensory neurons, actin-depolymerization drugs have no impact on virus entry [143,184]. Conversely, the microtubule cytoskeleton and long-range microtubule-dependent transport are very important for alphaherpesvirus infection, especially in regard to axonal transport. Incoming capsids are often seen accumulating at the MTOC prior to reaching the nuclear membrane, where they dock at nuclear pores and release genomic DNA into the nucleus [143]. Minus-end directed transport towards the MTOC requires the dynein motor complex, and dynein association with the vertices of incoming capsids has been detected by immunoelectron microscopy [143]. Disruption of the dynactin complex, a cofactor of microtubule motors including dynein [91,108], interferes with retrograde transport of incoming capsids [143,185]. High-speed time-lapse microscopy has allowed for tracking of individual fluorescently-labeled capsids in cultured sensory neurons, revealing that transport occurs by fast axonal flow (>1 μm/s), is saltatory, and bi-directional [177,186]. Bi-directionality indicates that capsids are simultaneously bound to microtubule motors of opposite polarity (*i.e.*, dynein and a kinesin), and the capacity for capsids to bind opposing motors was recently demonstrated (Figure 2B) [187]. The reason for recruitment of a plus-end directed kinesin on the incoming capsid is not immediately clear; however, the final transport of capsids from the MTOC to the nucleus may be achieved by a regulatory switch from dominant dynein activity to motion favoring kinesin. In fact, little is known regarding how herpesvirus capsids transit from the MTOC to nucleus, and whether this is a facilitated process. Because the MTOC resides in close proximity to the nucleus in many cells types, passive diffusion may be sufficient for this final step. Adenoviruses, which like herpesviruses transport to the MTOC and then dock at nuclear pores, actively sense nuclear proximity based on the nuclear export activity of the CRM1 (Chromosome Region Maintenance 1) protein [188]. Either a protein exported from the nucleus by CRM1, or CRM1 itself, is necessary for the MTOC to nuclear pore transition; whether a similar event occurs during herpesvirus infection has not been documented. A second possible role for kinesin recruitment on incoming capsids is in polarized epithelia. Because these cells have longitudinal microtubules oriented with minus ends apical, an incoming herpesvirus capsid may require kinesins to move toward the nucleus.

A number of HSV proteins interact with microtubule motors, including: pUL34, pUL35 (VP26), pUL46 (VP11/12), pUL56 and pUS11 [189–192]. Only one of these is known to remain associated with incoming viral capsids: the exterior capsid component VP26 [177,193,194]. When microinjected into Hep2 cells, capsids assembled in insect cells using recombinant baculovirus vectors display an enhanced predilection to redistribute near the nucleus when VP26 is present [190]. Opposing this finding, capsids isolated from infected cell nuclei fail to bind dynein and move on microtubules *in vitro* suggesting that capsid protein components, including VP26, do not contribute to retrograde transport [195]. These apparently contradictory findings are difficult to resolve by nature of the different assays used by the studies. However, because recombinant HSV-1 and PRV lacking VP26 move retrograde effectively in cell lines and in primary sensory neurons in culture and *in vivo* [196–198], the VP26-dynein link is at best a redundant mechanism and additional recruitment mechanisms must exist. Tegument proteins are likely candidates for tethers between capsids and dynein. Many of these proteins have been shown to be dispensable for dynein recruitment either genetically or biochemically, including: pUL4, pUL11, pUL13, pUL14, pUL21, pUL41 (VHS), pUL46 (VP11/12), pUL47 (VP13/14), pUL48 (VP16), pUL49 (VP22), pUL51, pUS3, pUS11, ICP4 and ICP34.5 [187,197].

By process of elimination, the best candidate viral proteins for dynein recruitment are two tegument proteins: pUL36 (VP1/2) and pUL37. The VP1/2 tegument protein is the largest protein encoded in the herpesvirus genome (>3000 a.a.), and has increasingly been a focus of investigation. The protein performs essential functions during initial and late infection. Incoming capsids require VP1/2 to release DNA into the nucleus upon docking at nuclear pore complexes [199,200], and outgoing progeny capsids require VP1/2 for assembly and final envelopment of infectious particles in the cytoplasm [201–203]. Because of the central role VP1/2 serves in virion assembly and its large size, the protein is likely a scaffold for the recruitment of other tegument, and, possibly, envelope proteins. To date, only two other viral proteins have been confirmed as VP1/2 binding proteins: The pUL25 capsid protein [204–206] and the pUL37 tegument protein [207,208]. Several additional VP1/2 binding proteins have been identified in yeast-two-hybrid screens, including: pUL17, pUL18 (VP23), pUL26 (VP24), pUL31 and VP16 [204,209]. In addition, a 150 kD protein that is likely VP5 (major capsid protein) co-immunoprecipitates with VP1/2 from infected cells [210]. VP1/2 is associated with capsids vertices via the pUL25 interaction that is possibly strengthened through additional interactions with the pUL17, VP5 and VP23 capsid proteins [204,205,211–213]. The interaction of VP1/2 with the capsid surface, and pUL37 with VP1/2, constitutes these two tegument proteins as components of the “inner” tegument. Deletion of VP1/2 or pUL37 in HSV-1 or PRV results in accumulation of unenveloped capsids in the cytosol, implying that both proteins are required for virion assembly [201–203,214,215]. Because VP1/2 is an essential herpesvirus protein, null mutants unfortunately cannot be examined for transport defects during initial infection. However, VP1/2 is essential for cytoplasmic transport of progeny capsids post replication, strengthening its candidacy as a microtubule motor recruitment protein [216]. The pUL37 tegument protein is essential in HSV-1 [217], but pUL37-null PRV maintain some residual propagation [215]. This has allowed for a direct study of the role of pUL37 in nuclear delivery of incoming PRV capsids, and a substantial defect was identified [218]. It may be of interest that pUL37 is not required for delivery of HSV-1 capsids to nuclei when infected cells are artificially fused with uninfected cells to form syncytia [219]. Assuming that delivery of capsids emerging from the infected nucleus to uninfected nuclei within the syncytium uses microtubule transport, then the pUL37 defect seen in PRV may be due to a deficiency in another early step of infection, such as overcoming the cortical actin barrier. Isolation of capsids from extracellular HSV-1 particles followed by salt extraction demonstrates that capsid/tegument complexes have the capacity to bind kinesin-1, kinesin-2, dynein and dynactin independently and via different sites, and that these interactions likely occur with proteins of the inner tegument [187]. However, motor recruitment by capsid proteins cannot be ruled out yet: Although capsids isolated from infected cell nuclei fail to bind microtubule motors, the capsid proteins could be post-translational modified in the cytosol. How dynein is recruited to cellular cargoes is not fully understood, and because functionally significant interactions with dynein have been difficult to pin down for any virus, it seems likely that this recruitment may not be by a single viral protein-dynein interaction.

##### 2.2.2.2. Assembly, Egress and Cell-to-Cell Spread

Herpesvirus capsids assemble in the nucleus. HSV-1 and PRV induce the formation of intranuclear actin filaments, and these may promote capsid access to the inner nuclear membrane. Intranuclear HSV-1 capsids display actin-dependent directional movement [220]. Moreover, rod-like structures thought to be made of actin are observed bridging capsids and primary envelopes, further suggesting a possible role for actin in inner nuclear membrane budding [221]. Although there is some debate regarding the pathway of capsid egress from the nucleus, the favored model states that membrane-bound capsids that reside in the lumen of the nuclear membrane fuse with the outer nuclear membrane and release naked capsids into the cytosol (de-envelopment) [222–224]. Naked capsids proceed to the site of secondary envelopment where final assembly takes place. The process of envelopment/de-envelopment/secondary envelopment is conserved between different groups of alphaherpesviruses [225]. Tegument acquisition around capsids occurs in at least two steps: inner tegument proteins bind to capsids in the cytosol, and outer tegument proteins associate with nascent trans-Golgi network (TGN)-derived membranes rich in viral glycoproteins and are acquired concomitantly during final membrane acquisition [226]. As will be discussed, there is evidence that cytosolic capsids participate in microtubule-based transport both prior and subsequent to secondary envelopment, and that these two transport steps likely occur by different mechanisms. The architecture of epithelial and neuronal polarity also places restrictions on how nascent virus particles must be targeted for infections to be productive. It is not known how viruses recruit motors, nor how the different stages of transport are targeted.

###### 2.2.2.2.1. Delivery to TGN and Secondary Envelopment

Up to the point of capsid egress from the nucleus to the cytosol, the infection has progressed through immediate-early and early viral gene expression, DNA replication, late gene expression, capsid assembly, and genome encapsidation. The cytosol has also undergone dramatic alterations since the incoming capsid traveled retrograde to the MTOC and nuclear pore hours before. It is in this modified environment that progeny capsids de-enveloping from the nucleus now reside. All structural proteins, including tegument and envelope constituents are present and ready for assembly.

The cytoplasmic assembly pathway has been dissected genetically and observed by electron microscopy. PRV deleted for pUL36 (VP1/2) lacks all evidence of tegument protein assembly on capsids, supporting the idea that VP1/2 is a scaffold for the assembly of additional tegument proteins [202]. PRV deleted for pUL37 remains competent to assemble VP1/2 onto capsids [208,215]. PRV deleted for pUL48 (VP16) or pUL3.5 remains competent to assemble both VP1/2 and pUL37 onto capsids [227]. In each of these three scenarios, naked capsids accumulate in the cytosol of the infected cell and generally fail to proceed to final envelopment. However, differences between these defective phenotypes have been noted. Cytosolic UL36-null capsids are dispersed, while cytosolic UL37-null capsids tend to stick together and form clusters. In contrast, the UL3.5-null capsids are less prone to aggregate and tend to localize near the Golgi network. These observations are consistent with a role of VP1/2 in capsid trafficking in the cytosol, with pUL37 either preventing capsid aggregation or performing an auxiliary role in the transport process. Live-cell fluorescence imaging of PRV capsids in infected cells reveals the highly dynamic nature of these viral particles [216]. Capsid transport is primarily dependent on microtubules although actin makes a small, but measurable, contribution [216]. Consistent with a role for VP1/2 in virus trafficking, fluorescent capsids expressed by PRV deleted for pUL36 (VP1/2) fail to transport along microtubules. Interestingly, the UL37-null exhibits decreased transport, but transport is not abrogated [216]. Because UL37-null PRV has a secondary-envelopment defect, this result indicates that naked capsids participate in microtubule transport during the egress phase of infection. The composition of the de-enveloped capsids may be similar, perhaps identical, to incoming capsids following entry: Both capsids are deposited in the cytosol and are bound to VP1/2, pUL37 and pUS3. As discussed, entering capsids are primed to recruit dynein, traffic to the MTOC, and continue to nuclear pores. Although speculative, it is possible that de-enveloped capsids emerging from the nucleus follow the same paradigm, but upon trafficking toward the MTOC become diverted by the viral tegument and glycoproteins accumulated in the Golgi/TGN. There are interactions between capsid-inner tegument complexes and membrane patches containing glycoproteins and tegument proteins. For example, an interaction between pUL21 (tegument, binds to capsids), pUL16 (tegument), and pUL11 (transmembrane) could trigger budding into the secretory pathway [207,228–231]. In this way, the de-enveloped capsids are removed from the cytosol and follow the trafficking pattern of the biosynthetic pathway to ultimately be exocytosed from the cell as an infectious virion. There is a recent suggestion that the VP26 capsid protein may assist in re-envelopment by binding the tetraspanin family member, CTMP-7 [232]. Several viruses (HIV, HCV, HTLV-1, *etc.*) use the tetraspanin family of proteins to bud out of infected cells [233].

In addition to binding cytosolic, VP1/2 and pUL37 can associate with outer tegument proteins independently of capsids. Localization of pUL37 to the TGN is dependent on VP1/2, which likely serves as a tether to the tegument proteins localized in the TGN membrane [234]. In fact, the VP16 tegument protein interacts with VP1/2 based on yeast-two-hybrid screen data, and VP16 is required for the incorporation of both VP1/2 and pUL37 into PRV light particles (viral particles released from cells that contain tegument and envelope but lack a capsid) [207,227].

###### 2.2.2.2.2. TGN to Plasma Membrane Transport in Epithelia

Delivery of enveloped virions newly budded into the TGN to the plasma membrane is the final step of virus egress leading to the exocytic event. The polar architecture of epithelial cells allows for apical, basal and lateral virion release. In general, apical release is expected to provide for transmission between hosts, while lateral release would promote viral dissemination within the epithelial sheet and basal release would deliver virions to nerve endings (Figure 2A). The unique orientation of microtubules in polarized epithelia predicts that kinesins would promote basal release and dynein apical release [66,180]. However, in HSV-1 infected epithelia in culture the majority of virions release laterally [235]. The polar release of virions does not appear to be a default pathway, but rather viral proteins likely remodel the secretory pathway as the infection progresses with the TGN dramatically redistributing to the lateral cell periphery [236]. TGN fragmentation and redistribution is dependent upon microtubules and is thought to enhance viral assembly [237–239]. How viruses become directed to release basally to allow for neuroinvasion is even less clear, but the lack of neural input in most tissue culture epithelia models could indicate that a neuron-epithelial junction is required for effective basal targeting in epithelial cells. When epithelial cells are co-cultured with neurons, HSV-1 readily transmits from epithelia to neurons [240].

HSV-1 virions in the TGN require protein kinase D (PKD) to release from cells [241]. PKD is a member of the diacylglycerol (DAG)-binding Ser/The kinases that are involved in fission of vesicles from the TGN [242]. Notably, PKD also directs basolateral targeting of small cargoes and therefore is likely important for epithelial-neuron transmission (*i.e.*, neuroinvasion) [243]. In a rat model, PRV neuroinvasion requires a deubiquitinase (DUB) activity embedded in the amino-terminus of the pUL36 (VP1/2) inner tegument protein while lateral dissemination remains intact with a DUB mutant virus [244]. Because DUB mutants infect neurons and spread within the rat nervous system equivalently to wild-type PRV following intracranial infection, the DUB mutant phenotype may be explained by a lack of basal release from epithelia, which would be in keeping with the observation that VP1/2 recruits kinesin to TGN-derived vesicles containing enveloped HSV-1 and promotes their transport along microtubules *in vitro* [245,246]. Although, VP1/2 is traditionally viewed as an inner-tegument protein and binds directly to the capsid surface via an interaction with pUL25 [205,206], there are several lines of evidence supporting that a subpopulation of capsid-free VP1/2 protein interacts with membranes during infection. First, VP1/2 is a component of L-particles [227,247]. Second, a fraction of VP1/2 is released from virions following extraction with non-ionic detergent [195]. Third, VP1/2 and pUL37 colocalize to the Golgi independently from capsids [234]. Therefore, VP1/2 may act apart from capsids to recruit kinesin to the outer (cytosolic) surface of cellular vesicles containing virions. Alternatively, capsid-bound VP1/2 may recruit kinesin to partially budded viral particles that are not fully closed [246]. The potential for interactions between VP1/2, PKD and kinesin are currently fodder for speculation, but these multiple findings provide initial glimpses into what is undoubtedly a complex viral rewiring of the secretory pathway. In keeping with this scheme, we have determined that VP1/2 binds to conventional kinesin (kinesin I) and directs TGN-derived vesicles to the cellular periphery [248]. In cultured hippocampal neurons, knockdown of PKD redirects transport of endogenous cargoes to axons whereas expression of a dominant negative PKD results in mixed transport to both dendrites and axons [249]. Herpesviruses could direct polar release by modulating PKD activity.

Microtubule transport in epithelia cells leading to polar release of virions may involve additional viral proteins that can bind kinesin motors. HSV-1 pUS11 binds KIF5B and KLC-like protein PAT1 [192,250]. However, pUS11 is a non-essential tegument protein that binds dsRNA and shuttles in and out of the nucleus, and is not conserved among members of *Alphaherpesvirinae*. HSV-2 membrane-associated pUL56 interacts with KIF1A (a kinesin-3 family member) and localizes to the Golgi and associated vesicles [189,251]. Although pUL56 is conserved in the neuroinvasive herpesviruses [13] it is also dispensable for virus propagation [252]. Ultimately, the microtubule transport of TGN-derived vesicles harboring virions to the plasma membrane is not sufficient for exocytosis: Myosin motors mediate the last step of alphaherpesvirus delivery to the plasma membrane by navigating the cortical actin cytoskeleton [253,254].

###### 2.2.2.2.3. Anterograde Axon Transport

After reactivation from latency, viral particles are sorted to axons from neural soma and transport to axon terminals where infectious particles disseminate. This anterograde-directed movement is crucial for successful re-infection of innervated tissue after reactivation from latency and successful spread between hosts. Whether virus components are simply passive cargoes that select the correct transport vesicle or actively promote transport to distal axons is not clear. In support of the latter, herpesviruses encode membrane proteins which contain sorting signals. In general, sorting signals can have two overlapping functions: targeting proteins to secretory vesicles and directing secretory vesicle traffic [255]. Sorting motifs have been studied in several herpesvirus membrane-anchored proteins. For example, anterograde transport of PRV virions in axons, as well as transport of gB, gC, gD and gE, is dependent on a classic Y-based sorting motif (YXXφ, where Y is tyrosine, φ is a large hydrophobic amino acid) in pUS9 [256,257]. Complementation of PRV pUS9 with EHV-1 and BHV-1 pUS9 suggested similar function of pUS9 in these viruses. Conversely, failure to complement PRV pUS9 with pUS9 from HSV-1 and VZV suggested that although pUS9 is highly conserved in alphaherpesviruses, functional differences exist between some homologs [258]. The role of lipid rafts in transport of viral particles is becoming more evident, not only for virus entry, but also for anterograde transport. HSV-2 pUL11 and pUL56, HSV-1 vhs and pUL11, and PRV pUS9, gB, gE and gD all associate with lipid rafts [259]. While the functional significance of these associations is not clear, PRV pUS9 association with lipid rafts is essential for pUS9-dependent axonal sorting [260].

Although virally-encoded membrane proteins participate in axon targeting of viral particles, whether this sorting occurs prior or subsequent to final capsid envelopment in the cytoplasm has been debated. Initial investigations of HSV-1 in axons by electron microscopy yielded results from several laboratories documenting fully-assembled virions within vesicles. Inoculation of HSV-1 into the footpads of mice allowed for the examination of DRG axons *in vivo*. Axons emanating from heavily-infected neural somas within DRG contained virions in vesicles [261]. Although this analysis was of proximal axons located within ganglia, two subsequent studies found virions in vesicles within myelinated regions of the spinal cord in a similar infection model [262], and in retinal ganglion cell axons following inoculation of HSV-2 into the vitreous chamber of the eyes of rabbits [263]. Together, these studies yielded similar results for HSV-1 and HSV-2 in different neuron types at either the proximal or mid axon using two different animal models. Similar findings with HSV were subsequently reciprocated in axons of the mouse trigeminal ganglion [264] and in rat sensory neurons in a two-chamber culture model [172]. These findings were challenged in 1994 by a study of HSV-1 in a two-chamber culture model using human neurons, which concluded that the anterograde transport of HSV-1 in axons occurred prior to final envelopment of the capsid: 100% of capsids in axons, both in proximal and distal regions, transported anterograde without envelopes [265]. The implication was that HSV envelope components must transport independently of the capsid to the distal axon where final envelopment would occur just prior to exocytosis (Figure 2B). Support for this model was gained by immunogold-EM [266]. Very recently, two additional laboratories examined this issue by conducting electron microscopic observations of HSV-1 infections in axons of cultured neurons: Both found predominantly enveloped virus particles resident in vesicles [267,268]. Similar studies have been conducted with PRV and have provided consistent support for envelopment preceding anterograde axon transport [269,270].

One reason that so much effort has been devoted to the issue of herpesvirus compositional state during axon transport is its relevance to understanding the mechanism of the transport process. If unenveloped capsids can transport anterograde in axons without membrane components, then capsids must recruit and activate kinesin motors to mediate this process. While there is good evidence that capsids/tegument complexes bind kinesin motors, it is not clear if these associations function to deliver capsids to distal axons during infection [187]. Two recent studies have attempted to address this issue by performing time-lapse imaging of recombinant HSV-1 strains encoding two fluorescent tags: one fused to the capsid surface and one to an envelope glycoprotein. Both studies confirmed that capsids co-transport anterograde with membrane components, consistent with the majority of electron microscopic studies reported for HSV-1. However, different conclusions were reached regarding the capacity for capsids to transport anterograde in axons prior to envelopment. In one case, a minority of capsids undergoing anterograde trafficking lacked detectable glycoprotein signal [177], while in the other glycoprotein signal could not be detected for the majority of these particles [271]. Detecting glycoprotein association with individual diffraction limited capsids is not trivial, and lack of glycoprotein detection could easily arise from limitations in the sensitivity of fluorescence emission detection during the short exposures (<1 s/frame) imposed by time-lapse imaging. Careful attention needs to be given to the imaging method, light source emission intensity, and detector sensitivity used in such studies. Additionally, because these studies used different recombinants of HSV-1, a side-by-side comparative examination of both recombinants should further help resolve this important issue. However, these approaches cannot easily rule out that a minority of capsids traffic anterograde in axons independently of the secretory pathway. While it is premature to conclude that capsids engage in long-distance axon transport by directly recruiting kinesin motors, the intriguing possibility remains.

Live imaging of fluorescently-tagged egressing PRV and HSV-1 viruses showed that both are co-transported with vesicle-associated membrane protein 2 (VAMP2), which is a part of the docking and/or fusion machinery of synaptic vesicles with the presynaptic membrane and a marker of axonal transport [272]. EM studies show that transport of HSV-1 viral glycoproteins and tegument proteins happens inside TGN-derived tubularvesicular membrane structures or large dense-core vesicles, and co-localizes with key anterograde traffic proteins: Rab3A, SNAP-25, GAP-43 and kinesin-1 [273]. These studies are beginning to unravel the aspects of the secretory pathway these viruses hijack for dissemination out of the nervous system.

High-speed time-lapse microscopy of fluorescently-labeled PRV capsids in dissociated cultured sensory neurons revealed that transport during egress occurs by fast axonal flow (∼2 μm/s), is saltatory, and bi-directional [274]. Remarkably, VZV reactivating in a 9-year-old boy was recently estimated to travel at 1.3 μm/s in the sciatic nerve [275]. The similar velocities reported in these two studies are perhaps even more remarkable in that the velocity reported for PRV was an average velocity of runs of continuous motion. These runs were punctuated by periods during which capsids were briefly stalled, attesting to the saltatory nature of the motion, that were not factored into the reported velocities. Therefore, the PRV and VZV velocities may be even a closer match despite one being measured in a tissue culture model and the other in a human leg.

#### 2.2.3. Viral Protein-Induced Cytoskeleton Rearrangements

There are a number of alphaherpesvirus proteins known to induce changes in the host cytoskeleton directly or through modulation of regulatory molecules. For example, HSV-2 anti-apoptotic gene ICP10PK influences survival of infected and adjacent neurons via the Ras signaling pathways MEK/ERK and PI3-K/Akt. Activation of these pathways causes neurons to release vascular endothelial growth factor (VEGF) and fractalkine [276], and stabilize microtubules via phosphorylation of microtubule-associated protein 1B (MAP1B) [277,278].

The actin cytoskeleton is affected by pUS3 kinase in PRV, BHV-1, and HSV [279–284]. pUS3, along with another viral kinase, pUL13, is also important for efficient TGN budding and proper anterograde transport [178]. pUS3 induces rearrangements in the actin cytoskeleton via activation of the PAK family of kinases, which leads to disassembly of actin stress fibers and induction of cellular protrusions, both of which are proposed to enhance cell-cell spread and viral egress [285]. Although attenuated in the natural host (pigs) [286,287], PRV US3-null viruses have only slight attenuation *in vitro* and in a mouse intranasal infection model, with delayed neuroinvasive symptoms [288]. HSV-2 pUS3 downregulates JNK/c-Jun phosphorylation *in vivo* [289], preventing virus-induced neuronal apoptosis in peripheral neurons and promoting spread of HSV-2 into the CNS [290]. HSV-2 US3-null viruses are attenuated in mice following either footpad or intraperitoneal inoculation, but only moderately attenuated following corneal and intracerebral inoculation [291,292]. Based on these findings, pUS3 was proposed to determine the neuroinvasive phenotype of HSV. In addition to induction of apoptosis, the JNK/c-Jun cascade regulates microtubule stability in axons by phosphorylating MAP1B, MAP2, tau, and other microtubule-associated proteins, which participate in regulation of microtubule stability in axons [293]. The interphase microtubule network is regulated by kinesin-1/JNK, which inhibits microtubule depolymerization, a novel function for conventional kinesin [294]. The effect of pUS3 on the actin cytoskeleton and possible regulation of the microtubule network via the JNK/c-Jun cascade are pro-survival, in line with the anti-apoptotic properties of pUS3 in a mouse intranasal model [295].

pUS3 also phosphorylates VP22, which is one of the most abundant tegument proteins [296–298]. Overexpression of HSV-1 VP22 induces stabilization and hyperacetylation of microtubules [299,300], which promotes motor binding and increases microtubule-associated trafficking [301,302]. VP22 interacts with mapmodulin (Golgi trafficking), template activating factors Ia and Ib (cyclin-dependent nucleosome and microtubule regulation), NMHC-IIA (non-muscular contractile myosin, HSV-1 entry receptor) [253], and microtubules [299]. In VZV, ORF9 (VP22 homologue) causes ORF66 (US3 homologue)-dependent redistribution of IE62 from the nucleus into the cytoplasm, where they co-localize with microtubules. Bovine herpesvirus VP22 (BVP22) has similar affinity for microtubules in the cytoplasm, but this association is less pronounced compared with HSV-1 and VZV VP22 [303]. Interestingly, HSV-1 VP22 and BVP22 are capable of inducing intercellular trafficking of nucleic acids and proteins between the cell of origin and untransfected or uninfected neighboring cells [304–310], which is a promising tool for enhancing neuron transfection efficiency for gene therapy [311]. Although the PRV VP22 homologue, pUL49, is dispensable for virus growth *in vitro* and had no neuroinvasion defects *in vivo* [312], HSV-1 VP22 is important for efficient viral spread both in cultured cells and mouse cornea, reducing HSV titers by one log in cultured cells [313].

There are other herpesvirus proteins known to affect the microtubule cytoskeleton. One of them is ICP0, which is a ring finger E3 ligase that regulates virus mRNA synthesis and the balance between replication and latency of the virus. In transfected or HSV-1 infected Vero cells, ICP0 translocates into the cytoplasm from the nucleus, where it bundles microtubules and dismantles them [314]. Another viral protein, pUL25, interacts with tubulin and ankyrin 2 (neuronal microtubule-associated protein) in a yeast-two-hybrid assay [315]. Additionally, pUL21, a binding partner for pUL16 and an important component of egress mechanism, binds to microtubules and causes the appearance of long processes when overexpressed in cultured cells [316]. Further studies of these HSV proteins *in vivo* could give insight on viral regulation of neuronal microtubule cytoskeleton.

## 3. Concluding Remarks

The neuroinvasive properties of the *Simplexviruses* and *Varicelloviruses* are unlike those of any other known viruses. While many viruses can invade the nervous system sporadically (*i.e.*, poliovirus) or following intramuscular puncture (*i.e.*, rabies), neuroinvasive herpesviruses are unique in their combined abilities to invade the nervous system routinely through undamaged tissue and regulate retrograde and anterograde trafficking effectively to enter and exit the nervous system. Chief among the tactics used by these viruses to effect this infectious cycle are numerous interactions with the cytoskeleton in two types of polarized cells: epithelia and neurons. The radically different cytoskeletal architectures of these cells are effectively navigated by these viruses by mechanisms that are only beginning to be unraveled. For a virus to spread from the apical surface of an epithelium to the nuclear pores of a neuron likely requires several reversals in microtubule transport direction along the way, which helps to explain why other viruses do not share the neuroinvasive program encoded by the herpesviruses. The complexity of the infectious cycle is matched by the complexity of these viruses. But with this complexity comes opportunities for the development of novel antiviral targets and scientific exploit. We have a lot to learn from a virus that can deliver genes to the nervous system merely by being placed on the skin, and it will only be a matter of time before we fully tame it. In the meantime, we can look forward to the continued identification and development of new classes of antivirals that exploit viral-cytoskeleton interactions [317].
